# Influence of pentylenetetrazol and NF-κB decoy oligodeoxynucleotides on p38 expression in neuron-like cells

**DOI:** 10.3892/etm.2014.1770

**Published:** 2014-06-10

**Authors:** JIA-JUN YANG, WEI-HUA LI, BANG-JIAN LIU, RONG-HUA TANG, YU-HONG ZHANG

**Affiliations:** 1 Department of Neurology, Sixth People’s Hospital of Shanghai Jiao Tong University, Shanghai 200233, P.R. China; 2 Department of Neurology, Tianyou Hospital, Wuhan University of Science and Technology, Wuhan, Hubei 430064, P.R. China; 3 Department of Neurology, Tongji Hospital Affiliated to Tongji Medical College, Huazhong University of Science and Technology, Wuhan, Hubei 430030, P.R. China

**Keywords:** pentylenetetrazol, NF-κB decoy oligodeoxynucleotides, neuron-like PC12 cells, p38

## Abstract

The aim of this study was to investigate the effects of pentylenetetrazol (PTZ) and nuclear factor κ B (NF-κB) decoy oligodeoxynucleotides (ODNs) on p38 expression in neuron-like PC12 cells. In addition, the role of NF-κB activation in the pathogenesis of epilepsy was explored. p38 expression levels in control and PTZ-treated neuron-like PC12 cells were examined using western blotting. NF-κB decoy ODNs were transfected into the neuron-like PC12 cells using Lipofectamine 2000. NF-κB activation was investigated by confocal laser scanning microscopy (CLSM), and p38 expression levels were assessed using western blotting prior to and following transfection of decoy ODNs. Western blot analysis revealed that p38 levels in PTZ-treated neuron-like PC12 cells were significantly higher than those in control cells. CLSM demonstrated that the decoy ODNs inhibited NF-κB activation in neuron-like PC12 cells, and western blotting indicated that the decoy ODNs did not reduce p38 levels. The results of this study indicate that PTZ enhances p38 expression levels and activates NF-κB in PC12 cells. However, NF-κB does not modulate p38 expression levels.

## Introduction

One of the directions actively pursued in epilepsy research is the reconstruction of hippocampal structural plasticity and function; this plasticity mainly manifests in synapses (1,2). p38 (also known as synaptophysin) comprises a pair of glycoproteins with a relative molecular weight of 38,000. p38 is expressed in the presynaptic vesicle membrane and it is associated with synaptic structure and function. As a presynaptic terminal-specific marker, p38 is often used to determine synaptic density and distribution. Variations in p38 expression are used to monitor the formation of synapses and the functional status of the nervous system. Due to the fact that p38 is not expressed by gliacytes, its expression reflects only neuronal plasticity. p38 expression has been studied in patients with epilepsy with the aim of evaluating synapse reconstruction and synaptic plasticity (3).

Nuclear factor κ B (NF-κB) is a dimer composed of two Rel family proteins. It is an important transcription factor that regulates the expression of multiple eukaryotic genes and influences a variety of cell functions (4). The activated forms of NF-κB are p50 and p65. When cells are in a quiescent condition, NF-κB binds to the inhibitory factor IκB, and the resultant protein complex remains in the cytoplasm. Following various cell stimulatory signals, NF-κB is activated by the phosphorylation and degradation of IκB or the phosphorylation of NF-κB through other IκB-independent pathways (5). These processes result in translocation of NF-κB to the nucleus, leading to regulation of the expression of downstream genes, including the immediate-early genes c-fos, c-myc and p53. Abnormal expression levels of c-fos, c-myc and p53 are associated with epileptic seizures (6,7). Blondeau *et al* (6) found that kainic acid was capable of facilitating the attachment of NF-κB to DNA, enabling one of the subunits of NF-κB to translocate to the nucleus. NF-κB activation is considered to be a key step in epileptic pathogenesis, and the role of NF-κB in epilepsy is presently the focus of numerous studies (8). It has been indicated that prior to pentylenetetrazol (PTZ) kindling or administration of a PTZ sub-dose to chronically stimulate epileptic seizures, NF-κB is activated to play an important role in epileptic plasticity (9). As a transcription factor, NF-κB participates in variations in epileptic plasticity by regulating the expression of multiple genes; such variations in epileptogenesis may be used to study the target genes of NF-κB. At present, whether NF-κB regulates p38 has yet to be elucidated.

PTZ is a central nervous system stimulant that induces acute and chronic kindling models of epilepsy, which may be used as model systems to investigate epileptic pathogenesis. The PC12 cell line is derived from rat adrenal phaeochromocytoma cells that are cultured in the presence of nerve growth factor to stimulate differentiation into neuron-like cells. Therefore, this cell line closely resembles neural cells in terms of morphology as well as physiological and biochemical functioning (3). The PC12 cell line is widely used as a model for physiological and pathological studies of neurons since neurons are difficult to culture *in vitro*. The aim of this study was to investigate the pathogenesis of epilepsy by exploring the molecular basis of variations in epileptic plasticity. In particular, the effects of PTZ and NF-κB decoy oligodeoxynucleotides (ODNs) on neuron-like PC12 cells and p38 expression were determined.

## Materials and methods

### Cell line

Rat phaeochromocytoma PC12 cells were maintained in Dulbecco’s modified Eagle’s medium (DMEM) containing 5% fetal bovine serum and 10% horse serum. The cells were cultured in an incubator containing 5% CO_2_ at 37°C with saturated humidity. The culture medium was replaced every two to three days. Neuron-like PC12 cells were prepared by exposure to nerve growth factor. The study was approved by the ethics committee of the Tongji Hospital Affiliated to Tongji Medical College, Huazhong University of Science and Technology (Wuhan, China). All reagents were purchased from Genemail Biotech Co., Ltd. (Xi’an, China).

### Grouping and cell treatment

Cells in the logarithmic growth phase were collected, adjusted to appropriate seeding concentrations with DMEM and inoculated into various culture plates (containing slides coated with polylysine). When the cells reached 70% confluence, transfection was conducted using Lipofectamine 2000, followed by a further 24 h of cell culture. The cells were divided into control (DMEM) and PTZ (final concentration, 10 mmol/l) groups. The PTZ group was subdivided into three groups: blank (without ODNs), missense NF-κB decoy ODNs and NF-κB decoy ODNs. The cells were then continuously cultured. NF-κB activity was determined using confocal laser scanning microscopy (CLSM) after 2 h of culture. In addition, MTT spectrophotometry was performed after 2 and 24 h of culture. The extent of cellular apoptosis was determined by flow cytometry and western blotting was conducted at 24 h.

### ODN sequences and transfection

The decoy ODNs (sequences: 5′-GAGGGGACTTTCCCT-3′ and 3′-CTCCCC TGAAA-GGGA-5′) were designed according to the sequence of NF-κB (4). A missense ODN (5′-GATGCGTCTGTCGCA-3′) and a control ODN (3′-CTACGCAGACAGCGT-5′) were also synthesized. Using these ODNs, transfection was conducted according to the manufacturer’s instructions.

### MTT assay

Cells were inoculated into a 96-well plate with a seeding density of 1×10^5^ cells/ml, prior to being divided and processed into the control and PTZ groups. The setup was performed in triplicate and a blank well was used as one of the controls. A total of 20 μl MTT solution (5 mg/ml) was added to each well and incubated for 4 h. Subsequently, the supernatant was removed and 100 μl dimethyl sulphoxide was added to each well. The cells were incubated for 20 h at 37°C. A Multiskan MK3 model automatic enzyme-labelled instrument was used to detect the absorbance values for each well. These absorbance values were indicative of the cell survival rate.

### Apoptosis assay

Cells were inoculated into a 96-well plate with a seeding density of 1×10^5^ cells/ml. The cells were divided and processed to form the control and PTZ groups, and the setup was performed in triplicate. The cells were washed twice with pre-cooled phosphate-buffered saline (PBS) and digested with 0.25% pancreatin to form a monoplast suspension. The monoplast suspension was centrifuged at 15,000 × g, fixed with 70% ethanol and washed with PBS to remove the fixative. The residue was reacted with RNase overnight. The resultant solution was mixed with propidium iodide (PI) liquid. DNA analysis was conducted using a flow cytometer.

### CLSM assay

NF-κB protein and a nuclear DNA fluorescent marker were inoculated into a 96-well plate containing 1×10^5^ cells/ml. The cells were grouped and processed to form the control and PTZ experimental groups. The cell slides were fixed with 4% paraformaldehyde, washed with PBS and sealed. p65 rabbit anti-mouse antibody (1:250 dilution) was added, and the cells were incubated at 4°C overnight prior to being washed with PBS. Fluorescein isothiocyanate (FITC)-labelled goat anti-rabbit antibody (1:50 dilution) was added, followed by incubation at 37°C for 30 min and the addition of 5 μg/ml PI plus 100 μg/ml RNaseA enzyme. The cell slides were protected from light for 25 min and mounted with glycerol for NF-κB detection.

The cell slides were viewed at 40× objective magnification. An argon ion laser was used as the light source and the excitation wavelength was 488 nm. The photomultiplier tube (PMT) 1 580 long pass emission filter was used to observe PI red fluorescence, and the PMT 2 522/DF35 emission filter was used to visualize FITC green fluorescence. The capacity factor was 30% and the scanning speed was slow. WinView/32 software was used to analyse the ratio of green fluorescence in the nuclear area (Fn) to red fluorescence in the cytoplasmic area (Fc). The Fn/Fc values of 20 cell samples were calculated.

### Western blotting

Cells were inoculated into a 96-well plate with a seeding density of 1×10^5^ cells/ml. The cells were grouped and processed to form the control and PTZ groups. Following culturing for 2 h, the cells were washed with ice-cold PBS and 50 μl cell lysis buffer was added. The resultant mixture was processed by ultrasonic wave four times (5 sec/round) and centrifuged for 15 min at 4°C and 12,000 r/min. The supernatant was recovered and the total protein concentration was determined using the Bradford method. The supernatant was stored at −70°C until further use. SDS loading buffer was mixed with the protein sample. The mixture was boiled in a water bath for 5 min and then stored at −20°C until further use.

SDS-PAGE was conducted at 8 V/cm and 360 mA for 90 min to determine the presence of p38. The protein was subsequently transferred onto a polyvinylidene fluoride film and sealed. Rabbit anti-human monoclonal p38 antibody (1:250 dilution) was added, prior to the mixture being incubated at 4°C for 16 h and washed with Tris-buffered saline with Tween 20. A horseradish peroxidase-labelled anti-rabbit immunoglobulin G secondary antibody (1:1,000) was added. After 1 h of incubation at room temperature, diaminobenzidine was added for development. Pan-actin was used as an internal reference. A gel imaging analysis system was used to document and analyse the gel data, and the p38/pan-actin absorbance ratio was calculated.

### Statistical analysis

All the experiments were performed in triplicate. Data are expressed as the mean ± standard deviation and analysis was performed using SPSS 12.0 software (SPSS, Inc., Chicago, IL, USA). Analysis of variance was used to compare the mean values.

## Results

### Cell survival rate

At 2 and 24 h, no significant differences were observed in the survival rate of neuron-like PC12 cells exposed to PTZ, missense NF-κB decoy ODNs or NF-κB decoy ODNs (pairwise comparisons, all P>0.05; Table I).

### Cellular apoptosis

**Figure 1:**
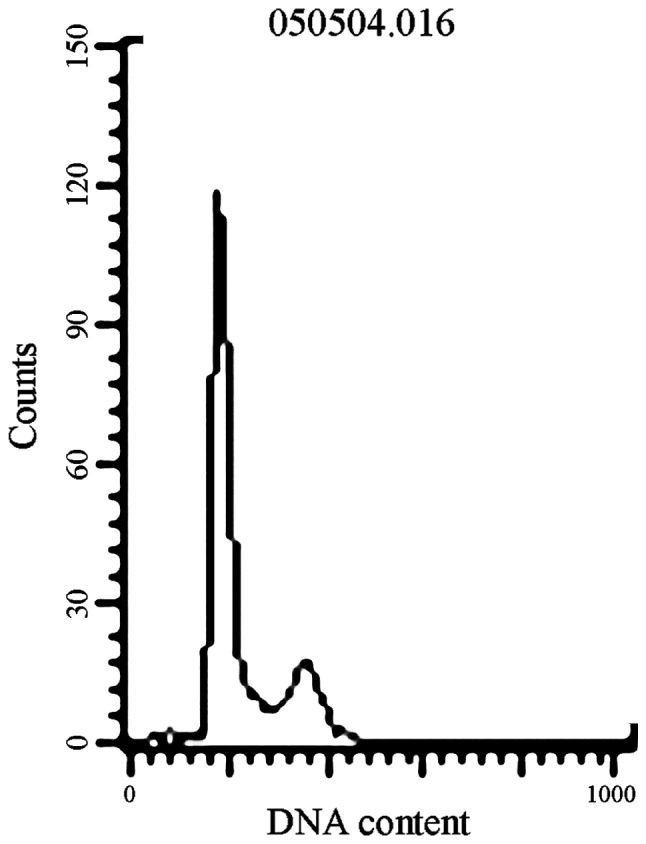
Effects of pentylenetetrazol, missense NF-κB decoy ODN and NF-κB decoy ODN on cell apoptosis. The horizontal axis represents propidium iodide fluorescence intensity, the vertical axis represents the number of cells, and each period can be distinguished from the fluorescence intensity. NF-κB, nuclear factor κ B; ODN, oligodeoxynucleotide.

The neuron-like PC12 cells in various groups exhibited no cellular apoptosis peak at 2 and 24 h (Fig. 1).

### NF-κB activity

**Figure 2:**
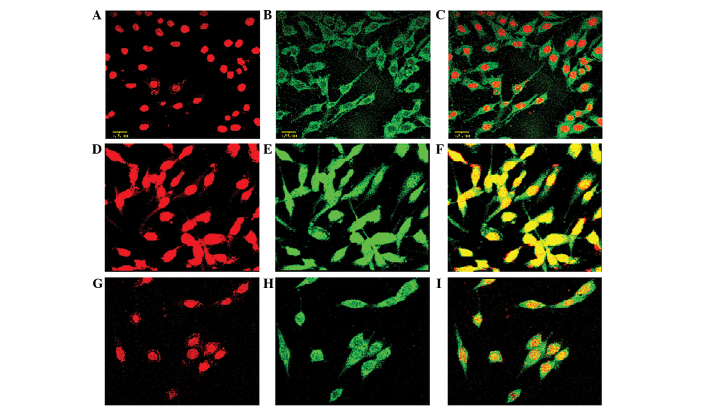
Neuron-like PC12 cells exhibited (A-C) round, red fluorescent areas (as shown by PMT1) and (D-F) green fluorescence representing p65 protein (as shown by PMT2). (G-I) Overlay of the PMT 1 and 2 images showed complete cell sections exhibiting a red nucleus and a green cytoplasm. (D) In the PTZ group, including the blank, missense NF-κB decoy ODN and NF-κB decoy ODN subgroups, PMT 2 showed that the whole cell exhibited green fluorescence and the intensity of green fluorescence was higher in the nucleus compared with the cytoplasm; (E) in the control group, including the blank, missense NF-κB decoy ODN and NF-κB decoy ODN subgroups, PMT 2 showed that green fluorescence was mainly distributed in the cytoplasm; (G-I) overlay of the cell images showed that the intensity of green fluorescence in the red fluorescent nuclear area of the PTZ group was significantly higher than that in the control group. PTZ, pentylenetetrazol; NF-κB, nuclear factor κ B; ODN, oligodeoxynucleotide, PMT, photomultiplier tube.

PMT 1 showed that the neuron-like PC12 cells exhibited round, red fluorescent areas (Fig. 2A–C), whereas PMT 2 showed that the neuron-like PC12 cells exhibited green fluorescence, representing the NF-κB p65 subunit (Fig. 2D–F). Once the two images were overlaid, a complete cell section was visible, with the nucleus in red and the cytoplasm in green (Fig. 2G–I). In the control group, PMT 2 showed that green fluorescence was distributed mainly in the cytoplasm (Fig. 2B). In the PTZ group, PMT 2 showed that the entire cell exhibited green fluorescence, which was more intense in the nucleus than in the cytoplasm (Fig. 2F and H). The overlaid images of the stained cells showed that the intensity of green fluorescence in the nucleus of the PTZ group was significantly higher than that in the control group (Fig. 2C, F and I). Statistical analysis revealed that the Fn/Fc ratio of the PTZ group was significantly higher than that of the control group (P<0.01). In the PTZ group, the Fn/Fc ratio of the NF-κB decoy ODN group was significantly lower than that of the blank and the missense NF-κB decoy ODN groups (both P<0.05). No significant difference in the Fn/Fc ratio was observed between the blank and the missense NF-κB decoy ODN groups (both P>0.05; Table II).

### p38 expression

**Figure 3:**
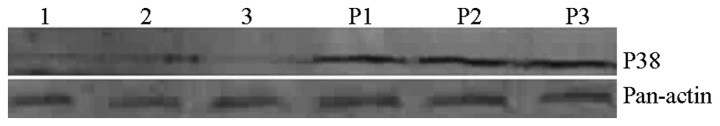
Effects of PTZ, missense NF-κB decoy ODN and NF-κB decoy ODN on p38 expression. Lane 1, blank/control group; lane 2, misssense NF-κB decoy ODN/control group; lane 3, NF-κB decoy ODN/control group; lane P1, blank/PTZ group; lane P2, missense NF-κB decoy ODN/PTZ group; lane P3, NF-κB decoy ODN/PTZ group. PTZ, pentylenetetrazol; NF-κB, nuclear factor κ B; ODN, oligodeoxynucleotide.

In the PTZ group, p38 expression was significantly higher than that in the control group (P<0.01). However, within the PTZ and control groups, no significant differences were observed among the blank, missense NF-κB decoy ODN and the NF-κB decoy ODN subgroups (all P>0.05; Table III; Fig. 3).

## Discussion

Biologists and medical scientists agree that the nervous system is characterised by plasticity. At present, one direction in epilepsy research is the reconstruction of hippocampal structural plasticity and function; this plasticity mainly manifests in synapses. p38 is a glycoprotein that is associated with synaptic structure and function. Changes in p38 expression are used to monitor the formation of synapses and the functional status of the nervous system. p38 is not expressed in gliacytes; thus, the expression of p38 reflects only neuronal plasticity. p38 expression has been used to study patients with epilepsy and animal models of epilepsy to evaluate structural changes due to synaptic plasticity (2,3).

PTZ is a central nervous system convulsant that acts a γ-aminobutyric acid receptor antagonist and does not cause neurotoxic effects. Exposure to successive sub-convulsant doses of PTZ leads to gradual apoptosis and necrosis of hippocampal pyramidal cells. Exposure to successive sub-convulsant doses of PTZ does not lead to apoptosis and necrosis of hippocampal pyramidal cells. PTZ is also an ideal compound for simulating generalised tonic-clonic epileptic seizures (10). Whether p38 expression changes prior to PTZ kindling has not been elucidated. Therefore, considering that PTZ does not affect the survival rate of neuron-like PC12 cells and does not induce apoptosis, the present study was conducted to examine the effects of PTZ on p38 expression and neuronal plasticity *in vitro* using neuron-like PC12 cells. No significant differences in the survival rate of neuron-like PC12 cells were observed among the groups at 2 and 24 h, and no apoptosis peak was observed in any of the groups. Under the experimental conditions, PTZ had no influence on the survival rate of neuron-like PC12 cells and apoptosis was not observed. At 24 h, p38 expression in the PTZ group was significantly higher than that in the control group, indicating that PTZ induces p38 expression. Chronic stimulation with sub-doses of PTZ may influence neuronal plasticity, potentially by modulating p38 protein expression.

NF-κB is a dimer protein composed of two Rel family proteins and its activation is a key step in epileptic pathogenesis. Animal studies indicate that the epileptic seizure-induced intracerebral inflammatory response is one of the main reasons for the pathological changes observed in the brain tissue of patients following epileptic seizures, particularly hippocampal structural damage (11,12). It has been suggested that the NF-κB signalling pathway has an important role in the expression and regulation of genes encoding cytokines and inflammatory mediators, and that overexpression of NF-κB may cause severe inflammation and tissue injury (13). A specific antagonist of NF-κB, pyrrolidine dithiocarbamate, inhibits epileptic seizures and intracerebral NF-κB expression in rats (14). Under the experimental conditions of the present study, the effect of PTZ on p38 expression and neuronal plasticity was examined, and NF-κB activity was determined using CLSM. PTZ was used to directly intervene in the functioning of neuron-like PC12 cells *in vitro*.

Wang *et al* (9,15) demonstrated that CLSM shows the location of NF-κB as well as its activation level on the basis of fluorescence intensities. An additional advantage of CLSM is that it shows cell morphology. The results of the present study showed that at 2 h, NF-κB activity in the PTZ group was significantly higher than that in the control group, indicating that PTZ was able to activate NF-κB. Therefore, it may be inferred that chronic stimulation with a sub-dose of PTZ affects neuronal plasticity, possibly by influencing NF-κB activity. Lubin (16) performed immunohistochemical analyses on brain sections obtained post-operatively from patients with temporal lobe epilepsy accompanied by hippocampal sclerosis. Overexpression of NF-κB was observed in gliacytes and pyramidal cells, indicating that epilepsy was induced by an NF-κB-mediated inflammatory reaction. This study also revealed that the inflammatory reaction was chronically active or transiently reinduced by repeated epileptiform seizures. Hippocampal neuron activation, in particular nuclear translocation of the p65 subunit of NF-κB, is an important mechanism of PTZ-kindled epilepsy formation in rats. Epileptic seizures are capable of inducing nuclear translocation of NF-κB in hippocampal tissue as well as interleukin-1β and cyclooxygenase-2 expression (17). PTZ increases protein expression of the p65 subunit of NF-κB in the brain tissue of rats with epilepsy (18,19). Another study indicated that epileptic seizures cause autophagic death of astrocytes via a pathway involving tumour necrosis factor-α and phosphorylated p65/RelA-Ser529 (20).

As a transcription factor, NF-κB contributes to variations in epileptic plasticity by regulating the expression of multiple genes. Therefore, studies on target genes being regulated by this transcription factor are important. One study indicated that insular epilepsy is closely associated with the hippocampus (18,19). This study also showed that growth-associated protein 43 and p38 are the pathological bases for this condition and the key molecular mechanisms in synaptic plasticity. Previous studies have shown that epilepsy-induced neuronal death is associated with various activations or deactivations of p38 and certain extracellular signal-regulated kinases (12,22). In particular, sesamin protects against kainic acid-induced brain damage in status epilepticus and inhibits the mitogen-activated protein kinase pathway through anti-inflammatory and partially anti-inflammatory mechanisms.

NF-κB and p38 participate in epileptic plasticity variation. As a presynaptic terminal-specific protein, p38 is a marker of synaptic plasticity; however, prior to this study the role of NF-κB in p38 regulation was yet to be elucidated. In the present study, an immunoblot assay was used to simultaneously determine p38 expression in neuron-like PC12 cells prior to and following exposure to PTZ, a missense NF-κB decoy ODN and an NF-κB decoy ODN. CLSM was used to assess variations in NF-κB activity. NF-κB decoy ODNs were artificially synthesised and competitively bound to the specific consensus sequence of NF-κB, reducing the binding of NF-κB to a target gene promoter and downregulating the double-stranded ODN expressed by the target gene. The decoy strategy has become a powerful tool for *in vitro* and *in vivo* studies of gene regulation due to the fact that it is associated with greater efficiency and selectivity than antisense ODNs (23). The missense NF-κB decoy ODN is a double-stranded ODN with a similar structure to that of the NF-κB decoy ODN. It differs in terms of key bases; thus, it is not able to competitively interact with the specific consensus sequences of NF-κB and has no regulatory effects on target genes. The results showed that following PTZ exposure, p38 expression in neuron-like PC12 cells was significantly increased. NF-κB activity in neuron-like PC12 cells decreased following NF-κB decoy ODN transfection; however, p38 expression levels remained constant. The missense NF-κB decoy ODN had no influence on p38 expression or NF-κB activity. These results suggested that NF-κB does not regulate p38 expression. Epileptic plasticity variation is a complex pathological process and further studies are necessary to determine the specific mechanisms involved.

## Figures and Tables

**Table I:** Influences of PTZ, missense NF-κB decoy ODN and NF-κB decoy ODN on the cell survival rate.

	Survival rate (A)			
	
	2 h		24 h	
		
Groups	Control group	PTZ group	Control group	PTZ group
Blank	0.402±0.074	0.426±0.053	0.411±0.067	0.420±0.042
Missense NF-κB decoy ODN	0.427±0.046	0.395±0.031	0.427±0.046	0.395±0.031
NF-κB decoy ODN	0.423±0.028	0.414±0.020	0.403±0.027	0.415±0.050

Absorbance (A) values are presented as the mean ± standard deviation. Pairwise comparisons of A values at 2 and 24 h, all P>0 05. PTZ, pentylenetetrazol; NF-κB, nuclear factor κ B; ODN, oligodeoxynucleotide.

**Table II:** Effects of PTZ, missense NF-κB decoy ODN and NF-κB decoy ODN on NF-κB activity.

	Fn/ Fc	
	
Groups	Control group	PTZ group
Blank	0.2161±0.0267	1.1063±0.1205^a^,^b^
Missense NF-κB decoy ODN	0.2312±0.0620	1.1067±0.1054^a^,^b^
NF-κB decoy ODN	0.2532±0.0527	0.7753±0.0774^a^

Fn/Fc values are presented as the mean ± standard deviation. Compared with the control group,

a P<0.01; compared with the NF-κB decoy ODN group,

b P<0.05.

PTZ, pentylenetetrazol; NF-κB, nuclear factor κ B; ODN, oligodeoxynucleotide; Fn, nuclear fluorescence intensity; Fc, cytoplasmic fluorescence intensity.

**Table III:** Effects of PTZ, missense NF-κB decoy ODN and NF-κB decoy ODN on p38 expression.

	p38/pan-actin	
	
Groups	Control group	PTZ group
Blank	0.2640±0.0496	1.0237±0.0724^a^
Missense NF-κB decoy ODN	0.2560±0.0614	1.0477±0.0705^a^
NF-κB decoy ODN	0.2493±0.0422	1.0390±0.0514^a^

p38/pan-actin values are presented as the mean ± standard deviation. Compared with the control group,

a P<0.01.

PTZ, pentylenetetrazol; NF-κB, nuclear factor κ B; ODN, oligodeoxynucleotide.

## References

[1] Eastwood SL, Burnet PW, McDonald B, Clinton J, Harrison PJ (1994). Synaptophysin gene expression in human brain: a quantitative in situ hybridization and immunocytochemical study. Neuroscience.

[2] Proper EA, Oestreicher AB, Jansen GH (2000). Immunohistochemical characterization of mossy fibre sprouting in the hippocampus of patients with pharmaco-resistant temporal lobe epilepsy. Brain.

[3] Davies KG, Schweitzer JB, Looney MR, Bush AJ, Dohan FC, Hermann BP (1998). Synaptophysin immunohistohemistry densitometry measurement in resected human hippocampus: implication for the etiology of hippocampal sclerosis. Epilepsy Res.

[4] Rauch BH, Weber AA, Braun M, Zimmermann N, Schrör K (2000). PDGF-induced Akt phosphorylation does not activate NF-kappa B in human vascular smooth muscle cells and fibroblasts. FEBS Lett.

[5] Schmitz ML, Bacher S, Kracht M (2001). I kappa B-independent control of NF-kappa B activity by modulatory phosphorylations. Trends Biochem Sci.

[6] Blondeau N, Widmann C, Lazdunski M, Heurteaux C (2001). Activation of the nuclear factor-kappaB is a key event in brain tolerance. J Neurosci.

[7] Savolainen KM, Loikkanen J, Eerikäinen S, Naarala J (1998). Interactions of excitatory neurotransmitters and xenobiotics in excitotoxicity and oxidative stress: glutamate and lead. Toxicol Lett.

[8] Lerner-Natoli M, Montpied P, Rousset MC, Bockaert J, Rondouin G (2000). Sequential expression of surface antigens and transcription factor NFkappa B by hippocampal cells in excitotoxicity and experimental epilepsy. Epilepsy Res.

[9] Wang KY, Ruan XZ, Zhang ZH, Zeng SQ (2001). Activation of nuclear factor κB assayed by laser scanning confocal microscope. Chinese Journal of Physical Medicine and Retabulitation.

[10] Qu H, Eloqayli H, Sonnewald U (2005). Pentylenetetrazole affects metabolism of astrocytes in culture. J Neurosci Res.

[11] Samland H, Huitron-Resendiz S, Masliah E, Criado J, Henriksen SJ, Campbell IL (2003). Profound increase in sensitivity to glutamatergic- but not cholinergic agonist-induced seizures in transgenic mice with astrocyte production of IL-6. J Neurosci Res.

[12] Voutsinos-Porche B, Koning E, Kaplan H (2004). Temporal patterns of the cerebral inflammatory response in the rat lithium-pilocarpine model of temporal lobe epilepsy. Neurobiol Dis.

[13] O‘Neill LA, Kaltschmidt C (1997). NF-kappaB: a crucial transcription factor for glial and neuronal cell function. Trends Neursci.

[14] Yu N, Di Q, Liu H, Hu Y, Jiang Y, Yan YK, Zhang YF, Zhang YD (2011). Nuclear factor-kappa B activity regulates brain expression of P-glycoprotein in the kainic acid-induced seizure rats. Mediators Inflamm.

[15] Wang KY, Ruan XZ, Zhu SQ, Wang W (2001). The role of the neuronal activation of hippocampus in epileptogenesis of pentylenetetrazol kindling rat. Journal of Apoplexy and Nervous Diseases.

[16] Lubin FD, Ren Y, Xu X, Anderson AE (2007). Nuclear factor-kappa B regulates seizure threshold and gene transcription following convulsant stimulation. J Neurochem.

[17] Wang SJ, Chi ZF, Wang SH, Chi LZ, Zhao XH (2010). Poly adenosine diphosphate-ribose polymerase regulates the expression of nuclear factor-κB and related inflammatory factors in rat hippocampus after epilepsy. Chin J Pathophysiol.

[18] Liu QZ, Wang F, Mu QC, Zhang PS, Sun T (2010). Expressions of GAP43, P38 mRNA and protein in insular electrical kindled rats and its significance. Zhonghua Yi Xue Za Zhi.

[19] Liu XW, Cai AM, Tian BX, Han K, Zhang XJ (2010). Study on activation of NF-κB p65 in rat hippocampal formation after experimental seizure and neuroprotective effects of W-7. Acta Universitatis Medicinalis Nanjing (Natural Science).

[20] Ryu HJ, Kim JE, Yeo SI, Kang TC (2011). p65/RelA-Ser529 NF-κB subunit phosphorylation induces autophagic astroglial death (Clasmatodendrosis) following status epilepticus. Cell Mol Neurobiol.

[21] de Lemos L, Junyent F, Verdaguer E (2010). Differences in activation of ERK1/2 and p38 kinase in Jnk3 null mice following KA treatment. J Neurochem.

[22] Hsieh PF, Hou CW, Yao PW (2011). Sesamin ameliorates oxidative stress and mortality in kainic acid-induced status epilepticus by inhibition of MAPK and COX-2 activation. J Neuroinflammation.

[23] Crinelli R, Bianchi M, Gentilini L, Magnani M (2002). Design and characterization of decoy oligonucleotides containing locked nucleic acids. Nucleic Acids Res.

